# *SHMT1 *1420 and *MTHFR *677 variants are associated with rectal but not colon cancer

**DOI:** 10.1186/1471-2407-10-525

**Published:** 2010-10-04

**Authors:** Viktor Komlósi, Erika Hitre, Éva Pap, Vilmos Adleff, Andrea Réti, Éva Székely, Anna Bíró, Péter Rudnai, Bernadette Schoket, Judit Müller, Béla Tóth, Szabolcs Ottó, Miklós Kásler, Judit Kralovánszky, Barna Budai

**Affiliations:** 1 School of PhD studies, Pathological Sciences, Semmelweis University, Budapest, Hungary; 2 Department of Clinical Research, National Institute of Oncology, Budapest, Hungary; 3 Medical Department, "Szent István és Szent László" Hospital, Budapest, Hungary; 4 Department of Cytogenetics and Immunology, National Institute of Chemical Safety, Budapest, Hungary; 5 Department of Environmental Epidemiology, National Institute of Environmental Health, Budapest, Hungary; 6 Department of Molecular Environmental Epidemiology, National Institute of Environmental Health, Budapest, Hungary; 7 Second Department of Pediatrics, Semmelweis University, Budapest, Hungary; 8 Department of Dermatology, Venerology and Dermatooncology, Semmelweis University, Budapest, Hungary

## Abstract

**Background:**

Association between rectal or colon cancer risk and serine hydroxymethyltransferase 1 (*SHMT1*) C1420T or methylenetetrahydrofolate reductase (*MTHFR*) C677T polymorphisms was assessed. The serum total homocysteine (HCY), marker of folate metabolism was also investigated.

**Methods:**

The *SHMT1 *and *MTHFR *genotypes were determined by real-time PCR and PCR-RFLP, respectively in 476 patients with rectal, 479 patients with colon cancer and in 461 and 478, respective controls matched for age and sex. Homocysteine levels were determined by HPLC kit. The association between polymorphisms and cancer risk was evaluated by logistic regression analysis adjusted for age, sex and body mass index. The population stratification bias was also estimated.

**Results:**

There was no association of genotypes or diplotypes with colon cancer. The rectal cancer risk was significantly lower for *SHMT1 *TT (OR = 0.57, 95% confidence interval (CI) 0.36-0.89) and higher for *MTHFR *CT genotypes (OR = 1.4, 95%CI 1.06-1.84). A gene-dosage effect was observed for *SHMT1 *with progressively decreasing risk with increasing number of T allele (p = 0.014). The stratified analysis according to age and sex revealed that the association is mainly present in the younger (< 60 years) or male subgroup. As expected from genotype analysis, the *SHMT1 *T allele/*MTHFR *CC diplotype was associated with reduced rectal cancer risk (OR 0.56, 95%CI 0.42-0.77 vs all other diplotypes together). The above results are unlikely to suffer from population stratification bias. In controls HCY was influenced by *SHMT1 *polymorphism, while in patients it was affected only by Dukes' stage. In patients with Dukes' stage C or D HCY can be considered as a tumor marker only in case of *SHMT1 *1420CC genotypes.

**Conclusions:**

A protective effect of *SHMT1 *1420T allele or *SHMT1 *1420 T allele/*MTHFR *677 CC diplotype against rectal but not colon cancer risk was demonstrated. The presence of *SHMT1 *1420 T allele significantly increases the HCY levels in controls but not in patients. Homocysteine could be considered as a tumor marker in *SHMT1 *1420 wild-type (CC) CRC patients in Dukes' stage C and D. Further studies need to clarify why *SHMT1 *and *MTHFR *polymorphisms are associated only with rectal and not colon cancer risk.

## Background

Colorectal cancer (CRC) is the second leading cause of cancer morbidity in Hungary both in male and female populations, and is one of the most frequent cause of cancer-related deaths. The mean age of the patients at the diagnosis of CRC is about 60 years and yearly about 9,000 new cases occur including approximately 2,800 newly diagnosed rectal cancer cases. Numerous results have been accumulated suggesting the role of folate and folate-related enzyme polymorphisms in the etiology of CRC. Low folate intake or low bioavailability of circulating folate, key components of one-carbon metabolism, have been related to increased risk of CRC [1]. The results, however, are still not conclusive. In a recent report an opposite influence of folate on different stages of the adenoma-carcinoma sequence was documented [2]. On the other hand, there are studies providing evidence for the significance of genetic polymorphisms of folate-cycle enzymes (e.g. methylenetetrahydrofolate reductase (MTHFR)) in colorectal carcinogenesis [3]. The role of the *MTHFR *C677T polymorphism influencing CRC susceptibility is also inconsistent [4,5]. In a meta-analysis of 25 studies Hubner *et al*. reported that *MTHFR *677TT genotype is associated with a significant, but moderately reduced risk of CRC [6]. Another less widely studied enzyme of folate/one-carbon metabolism, the cytosolic serine hydroxymethyltransferase (SHMT1), has a frequent but functionally less characterized Leu- > Phe polymorphism (SNP variant C1420T). This polymorphism was associated with significantly reduced risk of acute lymphocytic leukemia [7] and malignant lymphoma [8], but not colorectal adenoma [9]. However, in a CRC study no evidence of gene influence on the risk or association of this polymorphism with folate or homocysteine (HCY) levels were found [10]. The modifying effect of *SHMT1 *C1420T polymorphism on CRC risk remained undecided [11,12].

Rectal and colon cancer differ in their histology, diagnosis, sensitivity to radiotherapy and prognosis as well [13]. The treatment of rectal cancer usually includes preoperative radiotherapy, while colon cancer is non-radiosensitive. Both types of cancer have similar and also different risk factors [14,15]. Low folate and/or excessive alcohol intake are risk factors for both disease, but the physical activity have different effect on their risk [14]. In a large prospective US cohort body mass index (BMI) was related to increased risk of colon, but not rectal cancer [16]. There are few studies investigating the genetic risk factors separately in rectal and colon cancer, showing net differences between rectal and colon cancer [17-19]. The inconsistent results of the previous studies examining the polymorphisms in CRC may arise from the fact that dissimilar proportions of rectal and colon cancer cases were included in the investigations. Based on this information ascertained above the separate investigation of rectal and colon cancer cases is reasonable.

The SHMT1 is a vitamin B6 dependent enzyme that catalyzes the reversible conversion of serine and tetrahydrofolate (THF) to glycine and 5,10-methylenetetrahydrofolate (5,10-MTHF), a reaction which provides one-carbon units for S-adenosylmethionine (SAM), and for purine and pyrimidine synthesis.

Methylenetetrahydrofolate reductase irreversibly converts 5,10-MTHF to 5-methyltetrahydrofolate (5-methylTHF) using vitamin B2 as a cofactor. 5-methylTHF is the most stable and abundant form of folate metabolites, which in turn is the methyl donor in the conversion of HCY into methionine. Decrease in MTHFR reductase activity could lead to impaired HCY catabolism. There are two common functional SNPs on the *MTHFR *gene, which are in close linkage: the C677T and the A1298C substitutions. The C677T polymorphism on the exon 4 results in an Ala- > Val substitution at codon 222. Compared to the *MTHFR *677C homozygotes the TT and CT genotypes have 70% and 30% lower enzyme activity, respectively. The 677T allele has been associated with elevated plasma HCY levels [20]. In contrast, the exact functional consequence of the A1298C polymorphism is not well defined, and because of the linkage the extent of its independent contribution remains inconclusive [21,22].

Homocysteine is a sulphur-containing amino acid, produced mainly by S-adenosyl-methionine mediated methylation reactions. It is well-known that the total serum homocysteine (HCY) level among others is influenced by gender (higher in males), age (increasing with ageing), BMI (increased in obese individuals) and renal function (increased in renal impairment) [23-25]. Moreover, its accumulation in the serum is related to a number of disease conditions: vascular disease, cancer, neural tube defect, etc [26]. HCY reflects the combined pool of free, albumin bound, reduced and oxidized forms of HCY in the blood. Remethylation of homocysteine by methionine synthase, a vitamin B12 dependent enzyme, is the major metabolic pathway. The HCY level is inversely associated with the folate level and was suggested to be a risk factor of cancers [27,28]. Moreover a study suggested the role of HCY as a tumor marker of CRC [27]. This finding led to the exclusion of HCY from our analysis of cancer risk to avoid an eventual bias.

The aim of our study was to separately analyze the risk of rectal and colon cancer in association with *SHMT1 *C1420T and *MTHFR *C677T polymorphisms. The effect of the studied polymorphisms on the serum total HCY levels was also investigated.

## Methods

### Study population

For this case-control study 955 patients with colorectal adenocarcinoma were included. Cases of colon and rectal cancers were 476 and 479, respectively. The 461 and 478 sex and age matched healthy individuals recruited all across Hungary were considered as controls. Consecutive series of patients from all regions of the country diagnosed with rectal or colon tumors (Dukes' stage A, B, C and D) between 2001-2007 and who underwent surgery, radiotherapy and/or chemotherapy at the National Institute of Oncology (NIO) were included. This institute is one of the largest radiotherapy centre in Hungary therefore it treats higher number of rectal cancer patients than the average number treated elsewhere in Hungary. Out of the 24 patients with rectosigmoid lesion only 12, who ever received radiotherapy, were enrolled in the rectal cancer group. Patients with previous history of cancer or with synchronous cancer were not included in the study. All patients with hyperhomocysteinemia (HCY > 35 μM) possibly due to any renal impairment were excluded. The cases with proven or suspected familial adenomatous polyposis or hereditary nonpolyposis colorectal cancer were also excluded. The control population comprised healthy blood donors, healthcare workers or non-cancer patients from different regions of the country. The body mass index (BMI) was recorded both for controls and patients.

Written informed consent was obtained from all patients and controls before enrolment or sample withdrawal. The study was approved by the Ethics Committee of NIO.

### Preparation of the samples

Blood samples from patients and controls were collected after overnight fasting into EDTA (15%) containing tubes. Peripheral blood mononuclear cells (PBMC) were isolated from the whole blood by Ficoll gradient. DNA from PBMC was extracted according to the manufacturer's instructions using Master Pure TM Genomic DNA Purification Kit (Epicentre Technologies, Madison, WI, USA). The aliquots of blood plasma were frozen within 10 minutes after blood sampling and stored at -84°C for HCY determination according to the instructions of the HCY kit (Immundiagnostik AG, Bensheim, Germany).

### Genotyping and serum total homocysteine (HCY) determination

The *MTHFR *C677T (rs1801133) genotypes were determined by PCR-RFLP in conformity with the method described by Frosst *et al*. [29]. About 10% of the samples were re-examined by an investigator who had not attended the previous collection of data. There were no discrepancies in the results.

To determine the *SHMT1 *C1420T polymorphism (rs1979277) an allele discrimination method using fluorogenic 3'-minor groove binding (MGB) probes described by Skibola *et al*. was adapted [7]. The real-time PCR was performed in Rotorgene 2000 real-time cycler (Corbette Research, Mortlake, Australia). About 10% of the samples were parallel genotyped by real-time PCR and PCR-RFLP method using *Eam1104*I restriction enzyme (Fermentas Inc., Hanover, MD, USA). The discrepancy between the methods was below 1%.

The HCY level was determined by HPLC technique applying the HCY kit (Immundiagnostik AG, Bensheim, Germany). The interassay variability (overall coefficient of variation, %) of quality control samples was 4.4%. In patients the HCY levels were determined prior to surgical intervention and/or (radio)chemotherapy in fasting state.

During the above processes the investigators were blinded from sample categories.

### Statistical analysis

The means and proportions between cases and controls were compared by t test and χ^2 ^test for goodness-of-fit, respectively, except for HCY where Mann-Whitney U test was used. The difference in the distribution of diplotypes was analysed by pooling the groups with variant allele(s). Beside sex, the median age of 60 years was used as threshold for stratification. The association between polymorphisms and cancer risk was evaluated by logistic regression analysis adjusted for age, sex and BMI. In case of stratified analysis according to sex, age or BMI the adjustment was made for age and BMI, sex and BMI or age and sex, respectively. The results are given as odds ratio (OR) and 95% confidence interval (95% CI). Trends in the OR (gene dosage effect) were calculated by assigning ordinal values to the genotypes.

The Hardy-Weinberg equilibrium (HWE) was tested using the Haploview 4.1 software (Daly Lab at the Broad Institute, Cambridge, MA, USA).

The assumable potential impacts of population stratification bias in the studied population were estimated with the formulas of Lee and Wang [30]. The used formula is: U = (GB)^1/2 ^[(GB)^1/2^+1]^2^[(GB)^1/2 ^+G]^-1^[(GB)^1/2 ^+B]^-1^, where G = A_H_A_L_^-1^(1-A_L_)(1-A_H_)^-1 ^and B = D_H_D_L_^-1^; A_H, L _- highest and lowest allele frequency; D_H, L _- highest and lowest disease rate. The effective OR should be higher than the calculated U in order to have a result, which cannot be explained away by population stratification bias alone.

The *SHMT1 *C1420T genotype frequencies of European countries found in dbSNP [31], the *MTHFR *C677T genotype frequencies in Hungary presented in 12 independent publications and the colon and rectal cancer incidences in Europe [32] and in Hungary (National Cancer Registry for 2001-2007, I. Gaudi, personal communication) were used.

The age, sex, BMI and Dukes' stage adjusted HCY mean values in different geno- and diplotype groups were compared by Kruskal-Wallis ANOVA and Kruskal-Wallis Z post hoc test. The same analysis for different Dukes' stages was applied for HCY mean levels adjusted for age, sex and BMI. The linear trend was also tested for different Dukes' stages. The patients with Dukes' stage A, because of their low number, were included in the group with stage B.

All statistical tests were performed with NCSS software (Hintze, J. 2001. NCSS and PASS. Number Cruncher Statistical System, Kaysville, UT, http://www.ncss.com). A level of 5% or if the 95% CI did not include unity was considered significant.

## Results

Selected characteristics and the genotype distributions of patients and controls are summarized in Table 1. The HCY levels were significantly higher in cancer patients (mean ± SE: 20.8 ± 0.30; p < 0.0001 and 19.1 ± 0.27; p < 0.0001 for colon and rectal cancer, respectively) than in controls (17.9 ± 0.32 and 17.7 ± 0.30) (Table 1). The distribution of *SHMT1 *C1420T and *MTHFR *C677T genotypes in controls and cases was in conformity with the HWE (*SHMT1*: controls p = 0.131 and colon cancer p = 0.843; controls p = 0.167 and rectal cancer p = 0.973; *MTHFR*: controls p = 0.074 and colon cancer p = 0.063; controls p = 0.126 and rectal cancer p = 0.403).

**Table 1:** Selected characteristics and genotype frequencies of *SHMT1 *C1420T and *MTHFR *C677T polymorphisms of patients with colon cancer, rectal cancer and that of the respective controls

Parameters	Control	Colon cancer	p	Control	Rectal cancer	p
	n = 461	n = 476		n = 478	n = 479	
	n (%)	n (%)		n (%)	n (%)	
Age (years)						
mean ± SD	59.2 ± 12.4	59.5 ± 11.9	0.733	58.9 ± 11.1	58.9 ± 10.5	0.938
< 60	230 (50)	226 (47)	0.300	246 (51)	238 (50)	0.437
≥60	231 (50)	250 (53)		232 (49)	241 (50)	
Sex						
male	218 (47)	227 (48)	0.863	304 (64)	302 (63)	0.803
female	243 (53)	249 (52)		174 (36)	177 (37)	
BMI						
mean ± SD	26.2 ± 3.9	26.2 ± 4.0	1.000	26.5 ± 3.5	26.4 ± 4.3	0.882
Dukes' stage						
A		3 (1)			3 (1)	
B		107 (22)			102 (21)	
C		181 (38)			220 (46)	
D		185 (39)			154 (32)	

*SHMT1 *C1420T						
CC	220 (48)	228 (48)	0.360	220 (46)	249 (52)	0.002
CT	186 (40)	201 (42)		198 (41)	192 (40)	
TT	55 (12)	47 (10)		60 (13)	38 (8)	
CT+TT	241 (52)	248 (52)	0.939	258 (54)	230 (48)	0.009
*MTHFR *C677T*						
CC	216 (47)	208 (44)	0.396	226 (47)	190 (40)	0.002
CT	186 (40)	196 (42)		194 (41)	231 (48)	
TT	59 (13)	68 (14)		58 (12)	58 (12)	
CT+TT	245 (53)	264 (56)	0.230	252 (53)	289 (60)	0.001
*SHMT1 */*MTHFR*						
CC/CC	89 (19)	93 (20)	0.160	91 (19)	103 (22)	< 0.001
CC/CT+TT	131 (29)	132 (28)		129 (27)	146 (30)	
CT+TT/CC	127 (28)	115 (24)		135 (28)	87 (18)	
CT+TT/CT+TT	114 (24)	132 (28)		123 (26)	143 (30)	

Serum total homocysteine						
mean ± SD	17.9 ± 6.9	20.8 ± 6.5	< 0.0001	17.7 ± 6.6	19.1 ± 5.9	< 0.0001

* genotyping failed in 4 colon cancer patients

The univariate comparison of raw genotype distributions of cases and controls revealed significant difference only in case of rectal cancer for both *SHMT1 *C1420T and *MTHFR *C677T polymorphisms. An opposite distribution shift from variant homozygotes toward wild type and from wild type to hetorozygotes was present in case of *SHMT1 *and *MTHFR*, respectively, hence the *SHMT1 *CT+TT/*MTHFR *CC diplotypes were significantly underrepresented among cases with rectal cancer (Table 1). The distribution of unpooled diplotype groups is provided in the Additional file 1.

No association was observed in colon cancer for *SHMT1 *and *MTHFR *CT or TT genotypes compared with the CC genotype (Table 2). In contrast with colon cancer, the adjusted risk ratio for rectal cancer was significantly lower for *SHMT1 *TT and higher for *MTHFR *CT genotypes. A gene-dosage effect was observed only for *SHMT1 *with the progressively decreasing risk ratio with increasing number of T allele (p = 0.014).

**Table 2:** Overall, sex- and age-specific risk of colon and rectal cancer according to *SHMT1 *C1420T and *MTHFR *C677T polymorphisms

	Colon cancer	p	Rectal cancer	p
	OR* (95% CI)	p^†^	OR* (95% CI)	p^†^
*SHMT1*1420				
CC	1.00 (reference)	0.611	1.00 (reference)	0.014
CT	1.07 (0.81-1.41)	0.62	0.86 (0.66-1.13)	0.27
TT	0.86 (0.56-1.33)	0.49	0.57 (0.36-0.89)	0.013
CT+TT	1.02 (0.79-1.32)	0.90	0.80 (0.62-1.03)	0.08
*MTHFR *677				
CC	1.00 (reference)	0.338	1.00 (reference)	0.083
CT	1.08 (0.81-1.42)	0.61	1.40 (1.06-1.84)	0.016
TT	1.19 (0.80-1.78)	0.39	1.14 (0.75-1.73)	0.53
CT+TT	1.11 (0.87-1.44)	0.43	1.35 (1.04-1.74)	0.024

**Males**				
*SHMT1*1420				
CC	1.00 (reference)	0.334	1.00 (reference)	0.015
CT	0.93 (0.63-1.39)	0.73	0.92 (0.65-1.29)	0.63
TT	0.75 (0.40-1.39)	0.36	0.42 (0.23-0.75)	0.003
CT+TT	0.89 (0.61-1.30)	0.54	0.80 (0.58-1.10)	0.16
*MTHFR *677				
CC	1.00 (reference)	0.121	1.00 (reference)	0.494
CT	1.36 (0.91-2.05)	0.14	1.45 (1.03-2.05)	0.034
TT	1.42 (0.80-2.50)	0.23	0.89 (0.52-1.51)	0.67
CT+TT	1.37 (0.94-2.00)	0.10	1.29 (0.94-1.79)	0.12
**Females**				
*SHMT1*1420				
CC	1.00 (reference)	0.824	1.00 (reference)	0.390
CT	1.22 (0.83-1.80)	0.31	0.77 (0.49-1.22)	0.27
TT	0.94 (0.50-1.74)	0.83	0.97 (0.47-2.01)	0.93
CT+TT	1.13 (0.79-1.62)	0.50	0.80 (0.52-1.22)	0.29
*MTHFR *677				
CC	1.00 (reference)	0.863	1.00 (reference)	0.052
CT	0.87 (0.59-1.28)	0.48	1.39 (0.88-2.19)	0.16
TT	1.07 (0.60-1.90)	0.82	1.81 (0.91-3.60)	0.09
CT+TT	0.93 (0.65-1.33)	0.68	1.45 (0.94-2.23)	0.09

**Age < 60 years**				
*SHMT1*1420				
CC	1.00 (reference)	0.485	1.00 (reference)	0.002
CT	1.22 (0.82-1.82)	0.33	0.81 (0.55-1.19)	0.29
TT	0.67 (0.37-1.23)	0.19	0.32 (0.16-0.61)	0.0006
CT+TT	1.08 (0.74-1.57)	0.69	0.69 (0.48-0.99)	0.044
*MTHFR *677				
CC	1.00 (reference)	0.047	1.00 (reference)	0.112
CT	1.72 (1.15-2.59)	0.008	1.97 (1.33-2.92)	0.0007
TT	1.37 (0.77-2.44)	0.29	0.98 (0.54-1.76)	0.94
CT+TT	1.65 (1.14-2.39)	0.008	1.67 (1.16-2.40)	0.005
**Age ≥ 60 years**				
*SHMT1*1420				
CC	1.00 (reference)	0.943	1.00 (reference)	0.763
CT	0.91 (0.62-1.34)	0.64	0.90 (0.61-1.32)	0.58
TT	1.13 (0.59-2.18)	0.71	1.04 (0.54-1.98)	0.91
CT+TT	0.92 (0.64-1.33)	0.67	0.92 (0.64-1.31)	0.63
*MTHFR *677				
CC	1.00 (reference)	0.489	1.00 (reference)	0.432
CT	0.70 (0.47-1.03)	0.07	1.03 (0.70-1.52)	0.89
TT	0.99 (0.56-1.73)	0.96	1.33 (0.73-2.42)	0.35
CT+TT	0.75 (0.52-1.08)	0.13	1.08 (0.74-1.56)	0.70

* adjusted for BMI, sex and age for overall risk, BMI and age for sex-specific risk, BMI and sex for age-specific risk; † test of gene-dosage effect

The stratified analysis according to age and sex revealed that the association of rectal cancer with these polymorphisms was present only in younger (< 60 year) or male subgroups (Table 2). Interestingly, in case of colon cancer an opposite effect of the presence of *MTHFR *variant allele was seen for younger (OR > 1) and older patients (OR < 1), however, the association was significant only in the former case. In the stratified analysis according to the BMI (< 25 vs ≥25) there were non-significant differences only (data not shown).

Analyzing the association of cancer risk with diplotypes (Table 3) it was observed that the presence of *SHMT1 *T allele decreased the risk of rectal cancer only in case of wild type *MTHFR*. As had been expected from the results of genotype analysis the risk reducing effect of *SHMT1 *T allele was completely abolished when the *MTHFR *T allele was present. The ORs for all diplotypes is provided in the Additional file 1.

**Table 3:** Colon and rectal cancer risk based on *SHMT1 *1420/*MTHFR *677 diplotypes

Diplotypes	Colon cancer		Rectal cancer	
*SHMT1/MTHFR*	case/control	OR* (95% CI)	case/control	OR* (95% CI)
CC/CC	93/89	1.00 (reference)	103/91	1.00 (reference)
CC/CT+TT	132/131	0.95 (0.65-1.39)	146/129	0.99 (0.69-1.44)
CT+TT/CC	115/127	0.87 (0.59-1.27)	87/135	0.57 (0.39-0.84)^†^
CT+TT/CT+TT	132/114	1.10 (0.75-1.62)	143/123	1.02 (0.71-1.48)

* adjusted for age, sex and BMI; ^† ^different from reference (p = 0.005) or CT+TT/CT+TT (p = 0.002)

As the distribution of *SHMT1 *C1420T genotypes are not available for the Hungarian population the European genotype frequencies and rectal cancer incidences were used instead to gauge any potential population stratification bias. The *SHMT1 *1420 CC+CT frequency in Europe ranges from 0.833 to 0.913 [31], the rectal cancer incidence ranges from approximately 9 to 37 per 100 000 inhabitants in different countries in Europe [32]. Using the formula of Lee and Wang it was found that the upper bound for the bias is 1.29, which is less than 1/0.55 = 1.82, the estimated odds ratio observed in our study for carriers of *SHMT1 *1420C allele in Hungary. In the case of *MTHFR *C677T, we have found 12 independent publications for the Hungarian population, thus the range of CT+TT frequencies were in the range of 0.412-0.7. The rectal cancer incidence in Hungary ranges from approximately 32 to 38 per 100,000 individuals, thus the upper bound for the bias is 1.05, which is less than 1.38 found for Hungarian *MTHFR *677T allele carriers.

In order to investigate the effect of *SHMT1 *C1420T and *MTHFR *C677T polymorphisms the age, sex and BMI adjusted mean HCY levels in different diplotypes were compared in controls and patients. The HCY levels of patients were also adjusted for Dukes' stage, because the mean HCY levels were significantly higher in advanced stages of the disease (Table 4). In controls the presence of *SHMT1 *variant allele resulted in significantly higher HCY levels while this effect could not be observed in patients. The *MTHFR *T allele had no unequivocal effect on HCY levels. The adjusted mean HCY levels of all diplotypes are presented in the Additional file 1.

**Table 4:** Mean serum total homocysteine levels according to Dukes' stage and ***SHMT1 *1420/*MTHFR *677** diplotypes in colon and rectal cancer patients and respective controls

	Mean serum total homocysteine* (95% CI) [μM]			
Dukes' stage	Control	Colon cancer	Control	Rectal cancer
	17.9 (17.3-18.5)		17.7 (17.1-18.3)	
A, B		19.4 (16.9-21.8)		18.3 (16.9-19.6)
C		20.8 (19.1-22.5)°		19.1 (17.9-20.2)°
D		21.6 (20.7-22.6)°		20.4 (19.6-21.3)°
		p^† ^= 0.031		p^† ^= 0.036
		for trend p = 0.059		for trend p = 0.053
				
***SHMT1/MTHFR***	Control	Colon cancer**	Control	Rectal cancer**

CC/CC	15.6 (14.2-17.0)	20.7 (18.9-22.5)°	15.9 (14.6-17.2)	19.4 (18.0-20.8)°
CC/CT+TT	16.2 (15.1-17.4)	21.5 (20.1-22.8)°	15.3 (14.4-16.1)	21.0 (19.7-22.2)°
CT+TT/CC	19.1 (17.9-20.2)^#^	22.8 (21.0-24.5)°	19.3 (18.1-20.5)^#^	19.6 (17.9-21.4)
CT+TT/CT+TT	20.3 (19.1-21.6)^#^	21.4 (19.8-23.0)	19.8 (18.6-21.0)^#^	20.8 (19.7-21.9)
	p^† ^< 0.0001	p^† ^= 0.155	p^† ^< 0.0001	p^† ^= 0.221

* adjusted for age, sex and BMI; ** also adjusted for Dukes' stage; ^† ^Kruskal-Wallis one-way ANOVA;

^# ^different from CC/CC or CC/CT+TT, Z-value test p < 0.05;° different from control, Mann-Whitney U-test p < 0.01

## Discussion

In our study both investigated polymorphisms, *SHMT1 *C1420T and *MTHFR *C677T, exert influence on the risk of rectal but not on that of colon cancer. Previous studies underline the capacity of *SHMT1 *C1420T polymorphism being directly related to cancer susceptibility. Skibola *et al*. found risk reduction for the *SHMT1 *1420TT genotype in adult acute lymphocytic leukemia [7]. The same result was found by Hishida *et al*. in malignant lymphomas [8]. Recently, it was observed that in a North Chinese population there was a reduced risk of esophageal squamous cell carcinoma and gastric cardia adenocarcinoma in the case of *SHMT1 *1420CT heterozygotes compared to C homozygotes [33]. In the above mentioned studies the risk reduction influenced by the presence of *SHMT1 *1420T allele was in accordance with our findings regarding rectal cancer. The variant SHMT1 enzyme may result in decreased production of 5,10-MTHF and accumulation of tetrahydrofolate, although the exact biological effect of this phenomenon or the complete mechanism leading to carcinogenesis is not yet known.

In a case-control study van den Donk *et al*. could not demonstrate association between colorectal adenoma risk and *SHMT1 *C1420T polymorphism. Unfortunately, the authors performed a stratified analysis according to sex and not to the adenoma localization [9].

Chen *et al*. could not prove any risk-reducing effect of the variant type *SHMT1 *C1420T polymorphism in case of CRC [10]. In his study only male cases and male hospital-based controls were used from Caucasian-American populations, but the rectal and colon cancer patients were not separately analyzed, thus the risk modifying effect of the *SHMT1 *C1420T polymorphism might be obscured. Guerreiro *et al*. found an increased risk of CRC in case of *SHMT1 *1420 C allele, although the strength of their observation is limited by the small number of variant homozygotes (n = 9) and moreover, the rectal and colon cancer patients were analyzed together [11]. Steck *et al*. found a borderline statistically significant decreased risk of colon cancer in whites, but not African Americans, with the *SHMT *1420TT genotype as compared to the CC genotype. High folate intake reduced the risk of colon cancer in all genotypes. In this study the control group for whites deviated strongly from HWE (p = 0.0042) [12].

The presence of the *MTHFR *677T allele represented a higher rectal, but not colon cancer risk compared to CC homozygotes. The relationship of *MTHFR *C677T polymorphism and CRC risk based on the results of previous meta- and pooled analyses remains unclarified [34,35]. It has to be mentioned that most of the studies investigating the *MTHFR *C677T polymorphisms did not separate rectal and colon cancer patients [35]. Recently, Cao *et al*. found that in males, among *MTHFR *677TT genotype carriers, the OR for colon cancer was 2.42, but that of for rectal cancer was 0.52 [19]. Their result was similar to ours regarding males older than 60 years with ORs 1.4 and 0.9 for colon and rectal cancer, respectively. The differing genotype distribution of *MTHFR *C677T in Chinese and Caucasian, the different lifestyle, etc. may account for the more modest difference.

In accordance with the explanations raised by Guerreiro *et al*., - regarding the controversial results of previous published studies about the CRC risk modified by the interaction of folate intake and *MTHFR *677 polymorphism -, we accept the following statement: the low folate intake and the presence of *MTHFR *677T allele or TT genotype predicts if there is an increased risk of CRC, while on the contrary high/adequate folate intake results in a risk reduction if the variant allele is present [11]. In Hungary the folate intake is generally low [36], thus our result regarding the rectal cancer risk is in accordance with the previous statement, however, for colon cancer this relation can be found only in case of younger individuals (< 60 years). Iacopetta *et al*. demonstrated an increased proximal, but not distal CRC risk in the presence of *MTHFR *677T allele and low folate intake or older (≥ 65 years) individuals. Possible reasons for the discrepancy between their and our findings might include the classification of cancers as colon or rectal and the investigated Australian population consisted not only of Caucasians [37].

In the present study the combination of the *SHMT1 *1420CT+TT and *MTHFR *677CC genotypes was found to imply the lowest risk for rectal cancer. In a recent study an interactive influence of *MTHFR *C677T and *SHMT1 *C1420T polymorphisms in the risk of esophageal and gastric carcinomas was also observed [33]. Decreased activity of SHMT1 and the unaltered activity of MTHFR may result in a decreased amount of 5,10-MTHF available for pyrimidine synthesis. The combination with the highest risk is characterized by high SHMT1 activity and decreased MTHFR activity, which resulted in an increased availability of folate for pyrimidine synthesis. The impact of the examined polymorphisms on the DNA methylation is very complex. The decreased activity of SHMT1 may lead to a decreased DNA methylation through a negative feedback chain accumulation of intermediers and decreased 5-methylTHF production.

Our study also suggests the role of gender differences in the etiology of rectal and colon cancer. Female hormones may influence the susceptibility for second primary colorectal cancer [38]. Moreover, a statistically significant association was found between the DNA mismatch repair gene *MSH2 *-118T > C polymorphism and a strong family history of CRC and this association was seen only in female but not male CRC patients [39]. The age of onset of CRC may also be influenced by polymorphisms [40]. An age-specific association was also observed for colon cancer but not for rectal cancer in case of apolipoprotein E (*apoE*) polymorphism [41].

The association between BMI and colon cancer described in a previous large cohort and a pooled study [16,42] was not demonstarted in our analysis, which could be due to the insufficient statistical power. The above studies also demonstrated sex-specific and age-specific associations.

Gauging the population stratification bias for both polymorphisms it can be concluded that our findings are unlikely to be biased. However, it should be considered that in case of *SHMT1 *the genotype distributions and CRC incidence used for calculations were not available for Hungarian, but only for the European Caucasian population.

Homocysteine is produced during the methionine dependent DNA methylation. For its remethylation the product of the MTHFR enzyme, the 5-methylTHF, is essential. The less important metabolic transformation of homocystein implies cystathion beta-synthase or liver betain-homocysteine methyltransferase. Considering the important functional role of SHMT1 in the production of methyl group for multiple metabolic pathways any disturbance in the protein expression due to the polymorphisms could result in the reduction of the available one-carbon units that is also necessary for the remethylation of homocysteine. Geisel *et al*. found non-significant elevated HCY levels in the *SHMT1 *1420TT group in senior healthy subjects [43]. Chen *et al*. reported significantly higher HCY levels in case of *SHMT1 *1420 CT genotypes in a study including healthy male subjects [10]. Similarly, in our study the presence of *SHMT *1420 T allele significantly increased the HCY levels in controls. In relative young healthy individuals (20-40 years) Pereira *et al*. [21] and Baily *et al*. [22] found significantly higher HCY levels in *MTHFR *677 TT genotypes compared to the other genotypes. Almost the same result was found by Semmler *et al*. [44] with the exception that the above association could not be demonstrated for older individuals (> 55 years). This latter data support our results that in controls (mean age 59 years) there were no association between HCY levels and *MTHFR *677 polymorphism.

The influence of the investigated polymorphisms on HCY levels in case of patients may be obscured because of the much narrow distribution of HCY levels (coefficient of variation lowered by ~20%) than in controls. The effect is likely to be in a relationship with a yet unclarified HCY-increasing mechanism. Proliferating tumor cells appear to be the main cause of accumulation of HCY and thus, the development of hyperhomocysteinaemia in cancer patients [45]. The tumor marker character of the HCY, namely the elevated HCY levels in cancer patients [45-54], the elevated HCY levels in advanced clinical stages [49,53] and the usefulness of HCY to monitor therapeutic effects [48,49,54,55] has also been demonstrated. Moreover, HCY concentration followed CEA levels in CRC patients [55]. This effect might be strongly related to the malignant disease progression as in our study the HCY gradually increased with the lymph node involvement (Dukes' stage C) and further on with the presence of distant metastases (Dukes' stage D).

In our study it seems at a first glance that HCY in Dukes' stage C and D is a tumor marker as it was suggested by Wu *et al*. [27], but if the *SHMT1 *1420 polymorphism is taken into account then HCY can be considered as tumor marker only in case of wild (CC) genotypes. This fact remains valid even for patients of Dukes' D stage presenting the highest HCY levels (data not shown). Similarly, Battistelli *et al*. presented significantly elevated HCY levels compared to controls only in case of MTHFR 677 CC+CT genotypes [50].

An interesting question would be why the HCY levels of rectal cancer patients in case of the "lowest rectal cancer risk"-presenting diplotype are not significantly higher compared to that of the controls as it can be seen in case of colon cancers. Could an unknown HCY-lowering mechanism confer resistance in this context against rectal carcinogenesis? Based on present results, it might be supposed, that in case of the risk-reducing diplotypes the low HCY compared to controls is directly responsible for reduced risk. On the other hand the low HCY could be an ancillary result and other mechanisms may lead to the reduced risk. Recently it was demonstrated [56] that if the variant *SHMT1 *is localized in the nucleus only an impaired *de novo *thymidylate biosynthesis is assured. The thymidylate biosynthesis is further diminished by the wild-type-*MTHFR*-transformed and thus depleted 5,10-MTHF levels. The impaired thymidylate biosynthesis is unfavourable for cell proliferation. These results suggest that the association between the elevated HCY levels and the cancer risk is not obvious and first of all the "tumor marker" aspect of HCY needs to be underlined.

## Conclusions

This is the first study presenting the protective effect of *SHMT1 *1420T allele or *SHMT1 *1420 T allele/*MTHFR *677 CC diplotype against rectal cancer risk. *SHMT1 *1420 variant significantly increase HCY levels in controls but not patients. HCY could be considered tumor marker only in wild-type (CC) *SHMT1 *1420 CRC patients in Dukes' stage C and D. Higher HCY levels are characteristics of patients in advanced stages of the disease. Further studies need to be conducted to reveal the complex role of *SHMT1, MTHFR *and other folate enzyme polymorphisms in colon and rectal carcinogenesis. The importance of HCY level also need to be clarified.

## Competing interests

The authors declare that they have no competing interests.

## Authors' contributions

VK, VA and BB carried out the molecular genetic studies, ÉP and AR carried out the HPLC assays, VK, JK and BB participated in the design of the study and helped to draft the manuscript, EH, ÉS, AB, PR, BS and BT participated in the enrollment and conduct of the study, JM, SO and MK participated in study coordination and revised the manuscript, VK and BB conceived the study, participated in its design and statistical analyses. All authors read and approved the final manuscript.

## Pre-publication history

The pre-publication history for this paper can be accessed here:

http://www.biomedcentral.com/1471-2407/10/525/prepub

## Supplementary Material

Additional file 1 **Distribution, cancer risk and serum total homocysteine level of diplotypes**. This file contains the distribution, cancer risk (OR and 95% CI) and mean serum total homocysteine (95% CI) level of unpooled *SHMT1 *1420/*MTHFR *677 diplotypes. Click here for file
